# Plant Cell Walls Tackling Climate Change: Biotechnological Strategies to Improve Crop Adaptations and Photosynthesis in Response to Global Warming

**DOI:** 10.3390/plants9020212

**Published:** 2020-02-06

**Authors:** Ignacio Ezquer, Ilige Salameh, Lucia Colombo, Panagiotis Kalaitzis

**Affiliations:** 1 Dipartimento di Bioscienze, Università degli Studi di Milano, 20133 Milan, Italy; lucia.colombo@unimi.it; 2 Department of Horticultural Genetics and Biotechnology, Mediterranean Agronomic Institute of Chania (MAICh), P.O. Box 85, 73100 Chania, Greece; salameh@maich.gr (I.S.); panagiot@maich.gr (P.K.)

**Keywords:** abiotic stress, salinity stress in plants, high temperature stress in plants, drought stress in plants, photosynthesis, cell wall, carbon sink/source, plant development, climate change, biotechnological tools

## Abstract

Plant cell wall (CW) is a complex and intricate structure that performs several functions throughout the plant life cycle. The CW of plants is critical to the maintenance of cells’ structural integrity by resisting internal hydrostatic pressures, providing flexibility to support cell division and expansion during tissue differentiation, and acting as an environmental barrier that protects the cells in response to abiotic stress. Plant CW, comprised primarily of polysaccharides, represents the largest sink for photosynthetically fixed carbon, both in plants and in the biosphere. The CW structure is highly varied, not only between plant species but also among different organs, tissues, and cell types in the same organism. During the developmental processes, the main CW components, i.e., cellulose, pectins, hemicelluloses, and different types of CW-glycoproteins, interact constantly with each other and with the environment to maintain cell homeostasis. Differentiation processes are altered by positional effect and are also tightly linked to environmental changes, affecting CW both at the molecular and biochemical levels. The negative effect of climate change on the environment is multifaceted, from high temperatures, altered concentrations of greenhouse gases such as increasing CO_2_ in the atmosphere, soil salinity, and drought, to increasing frequency of extreme weather events taking place concomitantly, therefore, climate change affects crop productivity in multiple ways. Rising CO_2_ concentration in the atmosphere is expected to increase photosynthetic rates, especially at high temperatures and under water-limited conditions. This review aims to synthesize current knowledge regarding the effects of climate change on CW biogenesis and modification. We discuss specific cases in crops of interest carrying cell wall modifications that enhance tolerance to climate change-related stresses; from cereals such as rice, wheat, barley, or maize to dicots of interest such as brassica oilseed, cotton, soybean, tomato, or potato. This information could be used for the rational design of genetic engineering traits that aim to increase the stress tolerance in key crops. Future growing conditions expose plants to variable and extreme climate change factors, which negatively impact global agriculture, and therefore further research in this area is critical.

## 1. Introduction

The Earth is getting warmer and extreme climatic events are becoming more frequent. Heavy rainfalls followed by prolonged periods of drought have led to accelerated evaporation of soil moisture and accumulation of salts in the soil; salinity has become an increasingly serious problem. The release, into the atmosphere, of huge amounts of carbon dioxide (CO_2_) depends largely on the use of fossil fuels such as oil, natural gas, and coal [1,2]. Upon combustion, CO_2_ constitutes one of the main greenhouse gases, as it is able to intercept the infrared radiation reflected from the planet’s surface following solar radiation. This effect increases the atmospheric temperature in a similar way that a greenhouse functions. Global warming has a direct impact on plants and biodiversity [3,4]. The UN Convention on Climate Change (COP25) recently held in 2019, pinpointed the extreme emergency to find active, decisive, ambitious, and direct solutions for climate change [5,6]. Initially, plants fix atmospheric CO_2_ by the photosynthetic process to transform it into organic chemical compounds necessary for development and growth. Plant cells are surrounded by a strong polysaccharide-rich cell wall (CW) that shapes the overall form, growth, and development. CO_2_ fixation by plants counteracts the greenhouse effect but the key question is whether it is sufficient to control the overall global warming. 

### 1.1. Plants Are Necessary to Remove CO_2_ from the Atmosphere

**Figure 1:**
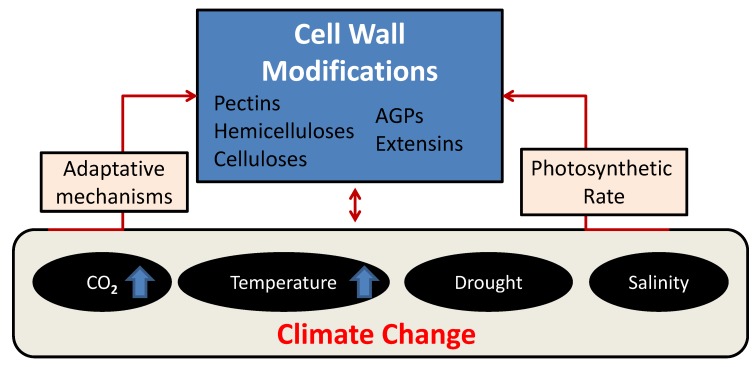
Diagram showing the interaction between climate change abiotic stress factors and plant cell wall (CW) responses. Climate change multifactorial abiotic stresses include drought, heat, salinity, and rise of atmospheric CO_2_. In addition, one of the consequences of rising CO_2_ concentration is expected to be the increase of photosynthetic rate, especially at high temperatures and under water-limiting conditions. The effects of global warming on CW homeostasis and adaptive mechanisms by CW reorganization to induce tolerance in crops are topics of discussion in this review.

The balance between the CO_2_ added into the atmosphere and that taken from the plants depends on multiple factors affecting the interconnections and energetic balances within an ecosystem, some of which are still poorly understood. For billions of years, bacteria, fungi, animals, and all non-photosynthetic organisms have consumed oxygen to respire and, consequently, have produced CO_2_ as waste. Plants, although they respire and consume oxygen, do the opposite, prevailing the photosynthetic activity on the respiratory one. Most of the fixed CO_2_ is transferred into the polysaccharide-rich CW of plants, alleviating anthropogenic increases in CO_2_ in the atmosphere by sequestering C in plant tissues and ultimately delivering it into soil organic matter. Therefore, understanding changes in CW homeostasis in response to global warming is absolutely necessary to develop strategies that mitigate climate change effects. The rising atmospheric CO_2_ has alerted the scientific community to the need to investigate any developmental alteration in the plants, given their direct influence on photosynthesis. Global warming increases leaf transpiration and induces water deficit in plants. The complexity of CW, in terms of composition and structure, suggests that CW synthesis and remodeling are controlled by an intricate regulatory network. Plants are the most effective organisms to tackle climate change by increasing net CO_2_ fixation via photosynthesis. Here, we review how photosynthetically fixed C, necessary for CW formation, responds to climate change-related environmental fluctuations. We describe how the global warming multifactorial effects such as rising atmospheric CO_2_ concentrations, increasing temperatures, as well as salinity and drought stresses influence plant developmental processes by remodeling CW in different plant tissues (Figure 1). A better understanding of these modifications would provide additional tools for breeders with regards to acclimation and adaptation of crops to combat climate change. The integration of both genetic resources and emerging molecular approaches such as genome editing and synthetic biology, together with ecological studies, have been applied to this aim [5,7].

### 1.2. Impact of Climate Change in Plants: Stress Responses and Adaptation

New strategies in plant breeding should focus on improving plant carbon uptake to increase plant biomass. The effect of climate change is multifaceted and the effects of these abiotic stress factors are synergistic and additive. To date, not enough has been done to meet the following three main goals: To reduce 45% of the emissions by 2030, to achieve climate neutrality by 2050 (thus a net zero carbon footprint), and to stabilize global temperature rise at 1.5 °C by the end of the century [1,8,9]. Therefore, it is urgent that scientific efforts be implemented that focus on global warming at different levels. First, by trying to mitigate the effects, for example, the use of plant systems to enhance CO_2_ fixation through the design of more efficient systems for CO_2_ fixation by plants that could counterbalance the greenhouse effect. We have reviewed strategies that aim to increase the rate of CO_2_ deposited into the CW of plants. This could counterbalance the rise in CO_2_ caused by human activities. Secondly, by adapting plants to the consequences. The plant science community is achieving significant efforts in the study of adapting mechanisms for crops [10,11]. This could facilitate adapting crops that are already growing in our agroecosystems and avoid the introduction of non-native species. We have reviewed how these detrimental effects impact CW modeling in plants and we have reviewed several cases where CW modifications have induced tolerance to several abiotic stresses linked to climate change. Leaf stomata function as valves that control the balance of water evaporation versus carbon fixation in plants, determining, in this way, crucial physiological processes necessary for plant life [12]. Recent findings have shown the impact of elevated temperature on anatomical traits of stomata, including modifications in cuticular waxes in these tissues. This review also discusses cellular aspects related to plant growth, photosynthesis, and the responses of stomatal development that arise due to chemical changes in the atmosphere, which are the causes of serious concern and discussion.

## 2. Discussion

### 2.1. Plant Cell Wall Modifications Are Coordinated to Developmental Processes 

Plant CW is a complex structure with critical functions in the plant life cycle. The CW maintains cell structural integrity by resisting internal hydrostatic pressures. The CW also provides flexibility by supporting cell division and expansion in proper balance to properly perform tissue differentiation. The CW also acts as a pathological and environmental barrier to protect the cells [13,14,15]. The CW incorporates a wide range of receptors and channels and controls the molecular trafficking and triggers specific responses to elicitors such as hormones, sugars, proteins, peptides, RNAs, and many other molecules. Consistent with the role of multiple biological processes, plant CW structure is especially varied between plant species, between tissue types, and between developmental processes. Cellulose forms nanoscale fibrils embedded in non-cellulosic matrix polymers. The mechanical properties of the CW depend largely on the integrity of the grid formed by the interlinked cellulose (fibrillary component) and the polysaccharide matrix. The morphological structure of plant cellulose fibrils such as the size, shape, and organization, are still not completely understood due to their heterogeneous nature and the limited resolution of the CW characterization techniques available nowadays. 

Developmental defects have been observed in cases of cellulose synthesis, and deposition is affected in seeds, roots, embryos, and among many other tissues [16,17,18,19]. The CW matrix is formed by other glucans, such as Xyloglucan (XG), a hemicellulose. XG remodeling drives proper cell expansion [20], CW integrity; XG metabolism, once compromised, impacts seed germination [21] and elongation processes (such as in fruits [22,23]). Pectins are the major CW matrix components of dicotyledonous plants. They are a heterogeneous family of polysaccharides formed by galacturonic acid (GalA) and rhamnose (Rha) [24]. Pectins are polymerized and methyl-esterified in the Golgi which are secreted into the wall as highly methyl-esterified forms. Subsequently, they can be modified by pectin methylesterases, which de-methylesterify homogalacturonan. The relationship between the esterified and the non-esterified pectins, and their distribution in the plant CW affect differentiation processes, contributing to cell cohesion and tissue firmness [25,26,27]. Pectins are involved in the formation of the pollen grain walls, as seen in *Quercus suber* L. and *Citrus clementina* [28]. Changes in pectin methylesterification occur in tomato, apple, and strawberry fruits during ripening; pectin methylesterification is also critical for proper seed development and germination [29,30], or root development [31,32]. The most abundant polysaccharides of the secondary CW are mannans, which play a key role for proper gynoecium, i.e., fruit transition [33,34]. 

#### 2.1.1. Following the Cycle of Carbon in Plants: From Carbon Fixation to Cell Wall Formation

**Figure 2:**
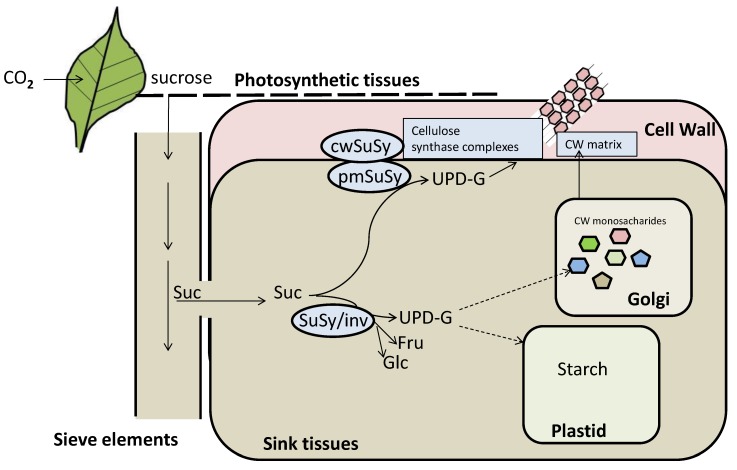
Photosynthesis is the source of carbon for CW synthesis. Fixed CO_2_ in leaves by photosynthesis in the chloroplasts is transported through the phloem and unloaded into the cytosol as triose phosphates. In growing sink organs such as meristems, leaves, roots, tubers, and seeds, most of the primary CW synthesis takes place using sucrose as the source of C and energy. Sucrose synthase (SuSy) and invertases are key enzymes controlling sugar precursors for CW synthesis [41,42,43,44]. Sucrose synthase catalyzes the reversible transformation of sucrose into fructose and UDP-Glc. Invertases catalyze the irreversible breakdown of sucrose into glucose and fructose. For a comprehensive view of starch synthesis see [45,46,47,48].

Nucleotide sugars, such as uridine diphosphate glucose (UDP-Glc), constitute the precursors for synthesis of CW polysaccharides [35]. Metabolic pathways of sucrose catabolism and provision of UDP-Glc for CW have been well reported [36,37,38]. CW synthesis requires sucrose as the source of C and energy (Figure 2). Several mechanisms for nucleotide-sugar formation contribute to cell wall synthesis [35]. As schematized in Figure 2, sucrose synthase (SuSy) and invertases play a tightly coordinated and fundamental role in CW formation because they are involved in the synthesis of precursor sugars required for cell wall components. CW polysaccharides are composed of a mixture of different sugars (not only glucose) and, prior to be incorporated into the polysaccharide matrix, these need to be activated to nucleotide sugars by specific post-translational modifications (such as redox regulation, phosphorylation, oligomerization, etc.) which reflect responses to climate change [35,39,40]. Therefore, climate change induced alterations of NDP-sugar supply to polysaccharide synthases could affect CW synthesis and structure. In addition to polysaccharides, the CW contains a variety of glycoproteins incorporated into the matrix with a structural and functional role. 

#### 2.1.2. Cell Wall Proteins Play a Key Role in Developmental Processes

One of the most abundant structural proteins in plant CW is the hydroxyproline-rich glycoproteins (HRGPs), which include several groups of O-glycoproteins. HRGPs include extensins, arabinogalactan proteins (AGPs), proline and hydroxyproline-rich proteins (P/HRGPs), and lectins [49,50]. HRGPs proteins play pivotal roles in CW-mediated signaling cascades, stress tolerance, and differentiation processes. They are incorporated into the matrix and are thought to provide further structural support in response to environmental changes. Several lines of evidence pinpoint that HRGPs generate an impenetrable physical barrier that is able to prevent pathogen infections [51,52,53]. AGPs are highly glycosylated members of the superfamily of hydroxyproline-rich glycoproteins (HRGPs) which are found in many species [54]. They contain a central protein backbone and a highly branched long glycan chain which is rich in galactose and arabinose [55,56]. In most conditions, glycosylphosphatidylinositol (GPI) anchor signals characterize the C-terminal of AGPs [57]. The action of AGPs has been reported in a wide range of plant organs (such as leaves, stems, roots, flowers, and seeds) with a fine-tuned spatiotemporally regulated expression [58,59]. AGPs are typically found in the plasma membrane, CWs, or in intercellular spaces, but are also present within intracellular and multivesicular bodies [55]. AGPs are involved in a multitude of developmental processes such as seedling growth [60], cell expansion [61,62], cell division [63,64], mechanical wounding [65,66], programmed cell death [67], and abiotic stress-response mechanisms such as salt tolerance [68], hypoxia, and anoxia [66]. Post-translational modifications cause profound changes in protein function. For example, proline hydroxylation, an early post-translational modification of HRGPs catalyzed by prolyl 4-hydroxylases (P4Hs), defines their subsequent *O-*glycosylation sites. P4Hs have recently been discovered to play an important role in plant development and growth [49,69]. Overall, differentiation processes involve multiple changes affecting CW both at the molecular and biochemical level. 

#### 2.1.3. The Interaction of Cell Wall Polymers in Developmental Processes

CW components interact constantly with each other and with the environment to control cell growth and maintain cell shape. However, it is still not clear exactly how the interactions between CW polymers relate to wall mechanics, and how transcription factors (TFs) impinge on intracellular structures during developmental processes in response to environmental signals. Many TFs such as MYBs, AP2/EREBP, WRKYs, MADS-box, or ERFs have been reported to play key functions in biotic and abiotic stress responses [10,70,71,72,73,74,75,76]. Developmental programs are regulated by complex networks both at the transcriptional and post-transcriptional level, including, not yet fully explored, epigenetic switches [73,77,78,79]. CW remodeling is a critical mechanism that copes with abiotic stress. The number of genes involved in CW synthesis, remodeling, and turnover in Arabidopsis is over 2000, suggesting that the CW continues to have a structural complexity that is not well understood [80]. The existence of a system of plant CW integrity maintenance in the primary CWs has been recently proposed [81]. Some phenotypes related to growth, development, and immunity described in several mutants or in some transformed plants with CW-modifying enzymes [82] are a consequence of the alterations of CW integrity sensing and signaling [13,83,84]. Evolution and crop domestication have forced plants to develop fine-tuned mechanisms to regulate biosynthetic pathways for CW components and have assembled them in a developmental program to allow the proper functioning of the cell [10,85,86,87,88,89,90,91,92].

### 2.2. The Impact of Climate Change-Induced High Photosynthesis in Cell Wall 

**Figure 3:**
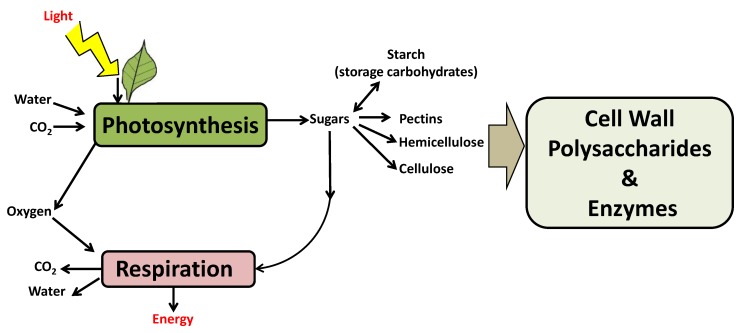
Plants fix atmospheric CO_2_ by the photosynthesis process to transform it into organic chemical compounds necessary for their development and growth. Schematic presentation of C metabolism in plant cells towards CW formation (structural role) or, alternatively, towards starch synthesis (storage role). CW biosynthesis is controlled by the photosynthetic rate and C status of the plant. During photosynthesis, light energy transfers electrons from water to fix CO_2_ in the chloroplasts via the Calvin cycle to yield sugars. C is directed to starch synthesis in the plastid or directed to CW formation, in the form of cellulose (fibrillar component), or to the CW matrix (pectin and hemicellulose) integrating other CW components such as proteins and lignin. This loop cycle is completed by respiration which occurs when sugars are combined with oxygen and generate useable cellular energy required for growth and normal cell functioning. CO_2_ and water are formed as by-products of respiration.

Climate change affects plants in many different ways, for example, by increasing photosynthetic rates in response to anthropogenic increases in atmospheric CO_2_ concentration [93,94]. Although some studies have investigated this phenomenon, the underlying basis for photosynthetic acclimation in response to climate change and the possible consequences of alterations in growth or developmental processes is not completely understood [95]. The plant CW controls the expansion and spatiotemporal distribution of cells in photosynthetic tissues. Cellulose biosynthesis in plants represents a huge carbon sink on the Earth. For example, the fibrillar component made of cellulose microfibrils forms a fundamental backbone of plant CWs and cellulose represents about 5% to 10% of plant dry weight. It is reasonable to think that cellulose production should respond to photosynthetic fluctuations by inducing changes in the availability of some precursors for biosynthesis or changes in the demand for CW deposition in response to growth fluctuations and organic C availability. Although there are some studies describing the relationship between the impact of photosynthesis and CW modulation, only a few evidences, so far, have shown that these parameters are mutually interconnected. Recently, it was shown that photosynthetic activity is a major regulator of cellulose synthesis and deposition by controlling both sucrose metabolism and cellulose synthesis complexes [96]. Interestingly, these authors showed that an increase in photosynthetic rates lead to a parallel increase of the C flux into cellulose synthesis and this was achieved via a tightly coordinated modulation in the phosphorylation of cellulose synthases (CesAs), as well as in other enzymes involved in sugar metabolism. Nevertheless, a complete picture of the role of cellulose biosynthesis in response to photosynthetic C provision is currently lacking. To date, there are no studies reporting the possible influence of alterations in photosynthesis rates and the modulation of other CW components such as pectins or hemicelluloses. However, based on the interconnections existing between C fixation capacity, it is reasonable to think that sugar homeostasis and CW deposition and photosynthesis could be mutually influenced (See Figure 3). It is noteworthy that biotechnological tools aimed at increasing biomass (CW) or starch accumulation have the potential to remove atmospheric CO_2_ and improve resilience to a changing climate.

#### Cell Wall Homeostasis Responds to Alterations in Photosynthesis

It has been shown that changes in leaf architecture is a result of deregulation of some genes involved in CW synthesis and reorganization [97]. Recently, it was demonstrated that changes in CW plasticity, due to changes in pectin composition, influence C partitioning by regulating the relationship between photosynthesis and plant growth [98]. Weraduwage and collaborators (2016) showed that suppression of pectin methylesterification in the double mutant *Golgi-related 2* and *3 (ggr2 ggr3)* reduced CO_2_ availability for photosynthesis. The link between photosynthesis, C homeostasis, and plant growth has already been established by studies of mutants that are defective in starch synthesis and mobilization. Starchless mutants impaired in those key enzymes involved in starch synthesis display a large inhibition of growth when cultured in short-day conditions, concomitantly, with reduced photosynthetic capacity [99,100,101,102,103]. Other players such as those controlling hemicellulose deposition could also be involved in partitioning of C toward leaf area growth. One of the major roles of xyloglucan endotransglucosylase/hydrolase (XTH) is the cleavage of cellulose and hemicellulose cross linkages which are necessary for wall loosening and cell expansion during elongation of tissues. In Arabidopsis, reduced expression of XTH8 and XTH31 affected leaf growth [104]. The leaf mesophyll in these mutants displayed a higher number of small mesophyll cells per unit area with respect to a control situation. In addition, suppression of XTH21 reduced cellulose and xyloglucan contents in leaves [105]. Shorter fruits and internodes were observed in Arabidopsis by disrupting α-xylosidase activity [21]. Therefore, it seems that hemicellulose remodeling and deposition play key roles controlling C partitioning to leaf area which affect overall plant growth and CW reorganization similar to the role played by GGR genes. Salinity stress and drought reduce the turgor pressure of the plant cells that stretch the CW to counterbalance physical stress by inducing changes in both the organization and interaction of the wall polymers. Under elevated atmospheric CO_2_ and heat stress, the expansive growth is co-regulated by the rate at which CW loosens and extends.

### 2.3. Cell Wall Remodeling under Salinity Stress

As mentioned before, salt stress reduces the turgor pressure of plants, and hence reduces growth; therefore, to counteract this effect, an increase in CW extensibility is required to maintain plant growth [106]. In fact, sodium ions influence pectin crosslinks and have also been reported to disrupt microtubule stability, and therefore influence cellulose deposition [107]. In Arabidopsis, the CesA complex member CesA8/IRX1 was reported to play a key role in drought and osmotic stress tolerance [108]. Mutation in one of the 10 Arabidopsis CesAs, results in enhanced osmotic stress tolerance. In Arabidopsis, SOS6, which encodes a CesA-like protein, was shown to play an important role in response to osmotic stress. In fact, *sos6-1* mutant is hypersensitive to salt stress and osmotic stress and showed an accumulation of stress-induced ROS in plant CW [109]. Yan et al. (2018) established a link between Pectin methylesterase31 (PME31) and salt stress tolerance in Arabidopsis [110]. They found that salt stress significantly increases PME31 expression and also reported that the knockdown mutant *pme31* displayed a hypersensitive phenotype to salt stress in seed germination and post-germination growth with lower expression of several salinity stress-related genes (*DREB2A*, *RD29A* and *RD29B*). An Arabidopsis mutant with reduced expression of a PMEI gene (*At*1g62760) exhibited reduced sensitivity to salt stress [111]. Phenotypes like enhancement of root growth, increase of fresh weight, and appearance of less chlorosis and necrosis in NaCl treatments in the *pmei* mutant, suggested that PMEI works as a negative regulator of salinity tolerance. In contrast, the overexpression of PMEI13 (*At*5g62360) in Arabidopsis induced tolerance to salinity stress. Transgenic lines presented higher rates of germination and enhanced root growth and overall viability when exposed to salt stress as compared with WT plants [112]. Pectin acetylesterase (PAE) modulate pectin acetylation on their C2 or C3 of galacturonic acid residues. The fine-tuning of the degree of pectin acetylation is important in establishing abiotic stress tolerance mechanisms [113]. In Arabidopsis, *PAE* genes are differentially regulated in response to diverse abiotic stress [114]. For example, in shoots, *AtPAE2* was highly induced in response to salt stress. In roots, *At*PAE4 expression was slightly overexpressed in response to salt stress as was *At*PAE8. A comparison of the CW composition in root tips from soybean (*Glycine max*) cultivars with different degrees of tolerance to salt stress showed more pectin deposition in the tolerant cultivar as compared with the sensitive line, suggesting that a higher accumulation of pectins in roots is beneficial for root growth in soils with high levels of salinity [115,116]. Shen et al., (2014) found that Arabidopsis plants which undergo salt acclimation could promote extensive remodeling of the CW, thus, priming and enforcing plants to be ready for subsequent salt stress [117]. In fact, at least 24 CW genes were differentially expressed during salt acclimation favoring CW loosening. For example, genes encoding for expansin, invertase GPRs, and AGPs were all upregulated under salt stress; while most genes involved in crosslinking of CW polymers or CW stiffening, such as pectinesterases and extensins, were downregulated. Balanced expansin activity is required for normal cell growth and wall remodeling [115]. In Arabidopsis, mutations in expansin-like gene *EXLA2* enhanced tolerance to salinity stress, and therefore less impact on inhibition of root growth as compared with WT was found in salinity stress [115,118].

Xyloglucan endotransglucosylase/endohydrolases (XTHs) play a role in CW loosening and enhance the tolerance to salinity stress by cleaving and linking xyloglucan molecules in primary plant CW. Increased XTH transcript levels were observed in plants exposed to multiple stresses [115,119]. Recently Han et al. (2017) showed that *Dk*XTH1 is involved in salinity stress tolerance in Arabidopsis [120]. Overexpression of *Dk*XTH1 triggered the formation of broader leaves as compared with WT plants and an increase in the rates of seed germination under high salinity [120]. In tomato (*Solanum lycopersicum*), the overexpression of persimmon *Dk*XTH1 gene caused larger cells, with higher density of CW and intercellular spaces via its involvement in CW assembly. In hot pepper (*Capsicum annuum*), *Ca*XTH3 was suggested to be involved in CW remodeling to strengthen wall layers [121]. The overexpression of *Ca*XTH3 significantly improved the tolerance to high salinity in both Arabidopsis and tomato plants [121,122]. In tobacco *(Nicotiana tabacum* L.), overexpression of the gene *Pe*XTH from *Populus euphratica* induced salt tolerance with respect to WT tobacco plants in relation to root and leaf growth and increased the percentage of plant survival under salinity stress [123]. 

#### Cell Wall Proteins Are Active Players in Response to Salinity

The release of different AGPs has been considered as an efficient response mechanism to salt stress in plants [124,125]. The involvement of AGPs in salt stress has been linked to the role of AGPs in controlling cell expansion. Recently, Olmos et al. (2017) performed several immunolabelling assays using different anti-AGP antibodies and reported that AGPs were strongly accumulated in the culture medium of salt-adapted tobacco cells [126]. In rice (*Oryza sativa*), combined microarray analysis and gene expression data showed the inhibitive effect of salinity over AGP-encoding genes such as *Os*AGP1 and *Os*AGP20 [127]. The presence of AGPs was also described in *Brassica oleracea* L. in which the xylem sap seemed to accumulate AGPs after 24 h of salt treatment [128]. In tobacco isolated cell cultures that resulted tolerant to NaCl-rich medium, cells displayed a reduced rate of cell elongation and a parallel decrease in CW extensibility [129]. NaCl-tolerant cells were deficient in AGPs on the plasma membrane. On the contrary, non-adapted tobacco cells showed high levels of AGPs on the plasma membrane [129,130] and an upregulation of AGPs in tobacco cells exposed to salt stress [68]. The role of AGPs expands, during salt stress, from an increase in the stiffness of the CW (when AGPs decrease), to the activation of signaling mechanisms by releasing extracellular AGPs, or to the accumulation of soluble sugars in the cytoplasm to decrease water loss [119,125]. The fasciclin-like arabinogalactan-proteins (FLAs) are members of the super-large family of arabinogalactan-proteins (AGPs). They are located at the cell surface and in the CW/plasma membrane. FLAs containing a single FLAS domain are important for structural conformation of the plant CW matrix (FLA11 and FLA12). FLA4/SOS5 plays a key role in maintaining proper cell expansion under salt stress conditions [131]. In Populus, 18 FLAs were expressed in root tissues under salt stress. Importantly, six of them, *Ptr*FLA2, *Ptr*FLA12, *Ptr*FLA20, *Ptr*FLA21, *Ptr*FLA24, and *Ptr*FLA30, were significantly induced at different time points which could indicate a FLA role in salt stress response [132]. FERONIA (FER), a plasma-membrane-localized receptor kinase, senses defects in pectin structure and wall elasticity caused by high salinity in order to activate signaling pathways [133]. It is required for maintaining CW integrity in root cells and activated growth recovery during salt stress [133]. For instance, salt stress likely disrupts both Ca^2+^ crosslinking of HG and borate-crosslinking of RG-II, which ultimately is sensed by FER; as a result, plant cells are able to maintain their CW integrity and recover growth without bursting [133]. Noteworthy, however, that the *fer* mutant presents constitutive expression of some typical CW damage responses and in the presence of high salt concentrations, root cells in *fer* mutants explode instead of recovering growth [134]. Other receptors-like kinase RLKs have been proposed to play a key role in the control of the integrity of cellulose. This is the case of LEUCINE-RICH REPEAT (LRR)-RLK, MALE DISCOVERER 1-INTERACTING RLK 2/LRR-KINASE FAMILY PROTEIN INDUCED BY SALT STRESS (MIK2/LRR-KISS), which is an important sensor of cellulose integrity in the wall; *mik2/lrr-kiss* mutants displayed increased sensitivity to salt stress, which could be associated with a diminished cellulose-sensing capacity [135].

### 2.4. The Effect of Elevated CO_2_ Concentrations and Cell Wall Modifications

Another aspect of the climate change is the elevated CO_2_ concentrations in the atmosphere. Primary CW is, thus, the first layer through which CO_2_ enters into the cell and acts as a limiting barrier controlling permeability of CO_2_ into the palisade layer [136]. Changes in CW thickness induce changes in mesophyll conductance that help plant adaptation to photosynthesis at high CO_2_ levels [136]. The CW is the major sink for carbohydrates on the Earth [137], thus, an excess of C input at elevated CO_2_ also alters CW composition. The development of the leaf area is an important component determining total plant productivity. In fact, leaf area increases in response to elevated CO_2_ conditions [138]. 

#### 2.4.1. Cell Wall Mechanical Properties and Structure Is Modulated in Response to High CO_2_

The expansive growth is co-regulated by the rate at which CW loosens and extends and by the rate at which water and solutes are accumulated by the growing cell [139]. Elevated atmospheric CO_2_ impacts *Quercus petraea* L. biomass production and CW composition of the leaves in favor of cellulose at the expense of lignin and enhances foliar non-structural carbohydrate levels and sucrose contents in a CO_2_ concentration-dependent manner [140]. Zhu and collaborators reported that CW thickness in mesophyll cells of flag leaves was statistically increased in rice and wheat (*Triticum aestivum*) when exposed to high CO_2_ at different growth stages [141]. Additionally, wall thickness was severely increased with elevated CO_2_ in mesophyll cells of Arabidopsis [142]. Several studies reported altered biochemical composition of CW in response to elevated CO_2_ [142,143]. An increase in cellulose content (approximately 33% in the leaves and 19% in the stems) was also observed in jatobá (*Hymenaea courbaril* L.) seedlings grown in an atmosphere with 720 ppm of CO_2_ [144]. In Arabidopsis leaves a similar increase by 22% was observed [142]. Elevated CO_2_ was shown to increase CW extensibility and stimulated cell expansion rates of roots and leaves possibly by an increase in the activity of XET [138]. However, the activity of xyloglucan endotransglycosylase XET solely could not promote loosening or extension of isolated CW and promote leaf growth in hoary plantain (*Plantago media*) under elevated CO_2_ [145]. Oksanen et al. (2005) reported a significant increase in hemicellulose contents in the CW of mesophyll cells in *Betula pendula* under high CO_2_. Although high CO_2_ conditions increased the CW dry weight, it did not affect CW composition, as observed in sweetgum (*Liquidambar styraciflua*) [146,147]. In barley (*Hordeum vulgare*) grown under high CO_2_ conditions, CW elasticity was significantly enhanced as compared with plants grown in normal CO_2_ levels [148]. This could indicate an adaptive mechanism that keeps cell turgor and prevents wall breakage while shoot elongation is stimulated by elevated CO_2_. Enhanced shooting was associated with an increase in the expression levels of both EXP and XTH, which are involved in the regulation of CW loosening during cell growth [119,149].

Sets of microRNAs have been shown to be important regulators of gene expression under elevated CO_2_ conditions in Arabidopsis. In fact, one of them, *mi*RPAL2, regulates key CW related genes such as cellulose synthases (*At*CesA10) and pectin biosynthesis genes (such as *GALACTURONOSYLTRANSFERASE-LIKE 9* (GT9) or *GLUCOSYLTRANSFERASE FAMILY 8* (GT8) [150]. These resulted upregulated under elevated CO_2_, suggesting that plant CW biosynthesis is modulated in response to environmental changes. In old aspen trees (*Populus tremuloides*), transcriptome analysis performed in high CO_2_ conditions showed a significant upregulation of genes encoding enzymes that control CW loosening and expansion like XTH or EXP [151]. This suggests the existence of a mechanism of CW remodeling in response to elevated CO_2_ that triggers radial growth expansion in perennial plants [152]. In aspen, a set of pectin-related enzymes were found to be upregulated in response to high CO_2_ conditions which included pectate lyases, pectin acetylesterases, and pectin methylesterases, and a the PME3 orthologous of Arabidopsis [119]. Similar deregulation was shown for several NDP-sugar epimerases [119]. These observations suggest that CW homeostasis changes (such as increasing the biosynthesis of pectins and hemicellulose) are triggered in vascular tissues during growth phases under high CO_2_ conditions. 

#### 2.4.2. Cell Wall Biosynthesis and Biomass Is Increased in Response to Elevated CO_2_

Regulation of substrate concentrations for CW biosynthesis could affect the ability of the plant to respond to CO_2_. Consistent with this, it was reported in Arabidopsis that elevated CO_2_ stimulates relative growth rate and leaf area gain with the effect of elevated CO_2_ in the UDP-Glc dehydrogenase activity [147]. Because the majority of the non-glucosyl sugars of the CW are derived from the interconversion of UDP-Glc beginning with the activity of UDP-Glc dehydrogenase, regulation of this activity could be critical in the synthesis of xyloglucan and other growth relevant matrix polysaccharides. In Arabidopsis, under high CO_2_ conditions, an increase in plant biomass by +20% was observed before flowering. The authors also found that UDP-Glc dehydrogenase, which is a key enzyme necessary for the UDP-Glc nucleotide interconversion for CW polysaccharide biosynthesis, increased [147]. Sucrose synthase (SuSy) is a glycosyl transferase that plays a key role in sugar metabolism, primarily in sink tissues. SuSy also directs C metabolic flow to cellulose synthesis, because SuSy catalyzes the reversible cleavage of sucrose into fructose and UDP-Glc, a substrate for both cellulose (β1-4) and callose (β1-3) glucans. Thus, SuSy plays a key role in the synthesis of both of these CW polysaccharides (Figure 2). It was reported that in *Opuntia ficus-indica* under twice as much CO_2_ concentrations increased sucrose synthase activity by 62% [153]. 

### 2.5. Cell Wall Remodeling under Drought Stress

Primary CW is highly hydrated (60% to 80% water) [154]. Drought impacts the water potential of the cell, and therefore turgor pressure stretches the CW to counterbalance physical stress by inducing changes in wall polymer structure and composition. The plant CW can be more flexible or rigid mechanically in order to control tissue growth [155,156]. In response, plants use multiple strategies to counterbalance drought stress via morphological and physiological changes, acting through diverse signaling cascades that lead to osmotic adjustments. Cellulose microfibrils, which are composed of ß-1,4-glucan chains, are major contributors in plant biomass formation, and their biosynthesis and accumulation is critical in order to induce plant defense against climatic fluctuations [157]. As reported in Arabidopsis, brassinosteroids signaling activates the *BES1* (*BRI1-EMS-Suppressor-1*) transcription factor, which binds to the promoter of CesA, induces its upregulation, and, in this way, triggers cellulose accumulation [158]. The CesA1 kinase activity has been shown to be activated upon the degradation of its inhibitor BRASSINOSTEROID INSENSITIVE 2 (BIN2) in drought conditions [159]. Introgression of the BR receptor containing the chromosome segment (7DL) from *Agropyron elongatum* triggered drought tolerance in wheat [160]. Expansins are known CW proteins that regulate CW loosening [161] by loosening the linkages formed between cellulose microfibrils [162]. Overexpression of *Ammopiptanthus nanus* expansins, *An*EXPA1 and *An*EXPA2, enhanced transgenic Arabidopsis tolerance to drought stress [163]. In fact, all transgenic lines were treated with water shortage, after water shortage for seven days, most of the WT plants did not survive and almost all the leaves were withered. However, *An*EXPA1 and *An*EXPA2 overexpressing transgenic lines displayed strong drought tolerance and higher survival rates. These results suggested that both *An*EXPA1 and *An*EXPA2 are involved in drought tolerance of plants by regulating CW loosening. Alterations in the expression of expansins led to enhanced resistance to abiotic stresses, and often this was accompanied by changes in plant growth and development [164]. For example, the expression of a wheat β-expansin (*Ta*EXPB23) in tobacco lead to drought resistance as compared with WT plants [165]. In soybean, the EXPB2 gene presented higher expression under water stress and was involved in improving root tolerance to drought stress [166]. In a similar way, in rose, the silencing of NAC2 transcription factor and the downstream target EXPA4 resulted in reduced drought stress during the development of the petal [167]. In addition, Arabidopsis plants overexpressing *Rh*EXPA4 displayed drought tolerance and presented shorter stems, more compact inflorescences, and curly leaves [167]. 

Pectins are often modified in plants exposed to drought stress. *Ca*PMEI1 from pepper is transcriptionally induced by drought stress [168]. Arabidopsis lines overexpressing *Ca*PMEI1 exhibited an increased tolerance to water stress, as shown by improved germination rate and seedling root growth as compared with control plants [113]. A comparative study was conducted that focused on the CW in wheat cultivars with different degrees of tolerance to drought stress [115]. Interestingly, in the tolerant cultivar, a higher level of side chains of the main pectinic components, rhamnogalacturonan I and II, were reported in response to drought stress. The authors proposed that a more ramified pectin backbound could facilitate the formation of hydrogels capsules that would limit the cell damage [169]. Recently, it has been demonstrated that pectin methylesterase gene *PME35* (*PtoPME35*) from Poplar (*Populus tomentos)* modulates stomatal function to provide drought tolerance. Yang and collaborators have shown that overexpression of *PtoPME3* in Arabidopsis inhibits stoma opening and this results in lower water evaporation in leaves under drought conditions [170].

Xyloglucan is the most abundant hemicellulosic polysaccharide in the primary plant CW. XTHs participate in xyloglucan metabolism by XET and/or XEH activities [171,172]. XTH activity induces CW to loosen or to strengthen [173]. Overexpression of *Ca*XTH3 has been shown to induce abnormal leaf morphology and severely wrinkled leaf shapes which improved tolerance to drought in transgenic Arabidopsis and tomato plants [121,122]. Arabidopsis plants overexpressing *Dk*XTH1 had significantly increased levels of seed germination under drought stress as compared with WT [120]. Glycoside hydrolases (GHs) are CW-related enzymes involved in the remodeling of the CW structure by organizing cellulose–cellulose interactions controlled by xyloglucans affecting CW extensibility [174,175] to facilitate plant cell expansion, differentiation, maturation, and the repair of the damaged wall under certain stresses [176]. The data of Landi and collaborators showed that in rice (*O. sativa* cv. *Nipponbare*) under drought stress at least two GH genes are activated in roots [177]. Noteworthy, an increase in UDP-Glc levels (the cellulose substrate) by overexpressing *SuSy* and UDP-glucose pyrophosphorylase (UGPase) encoding genes was shown to induce tolerance to water stress in concomitancy with enhanced cellulose accumulation [119]. 

In Arabidopsis, among the genes differentially regulated in response to drought, three pectin esterases, a pectin methyl esterase, and an AGP were identified [178]. An increase in the accumulation of these proteins helps preserve proper water levels in the wall in order to facilitate an optimal CW mechanical integrity in case the water levels fall below a certain critical level. This reorganization could also be implicated in the occurrence of a resistance mechanism, whereas the creation of a “buffer zone” prevents the direct interaction between membranes and CW matrix [125]. In rice, expression studies have shown that the expression of several AGPs such as *Os*AGP1, *Os*AGP15, and *Os*ELA3 was induced, in response to drought [179]. In addition, in rice, expression analysis in roots revealed genes differentially expressed in drought-resistant versus drought-sensitive genotypes, among which were identified some AGPs [180]. 

### 2.6. The Effect of Elevated Temperatures in Cell Wall Reorganization

Climate change constitutes a significant detrimental stress to plants. According to the Intergovernmental Panel on Climate Change Prediction, an increase of 2 to 5 °C, with respect to the current temperatures, is expected by the late twenty-first century [8]. The dramatic effects of high temperature produce multiple economical loses for farmers by dramatically affecting crop production. Nevertheless, plant cells have developed intricate systems in order to respond to a variety of challenges, including heat stress. The cellular damage imposed by heat stress causes protein denaturation and modifies aggregation of protein complexes by alterations in membrane permeability and fluidity. In addition, modified CW mechanical properties and composition affect the internal balance of metabolic processes (such as carbon sink/source balances). In nature, heat stress conditions are of variable duration [181]. Thus, during plant evolution and adaptation, and also with plant domestication, plants have acquired diverse systems to activate certain time-lapse high-temperature-related defense response mechanisms to prevent cell damage and protein aggregation [182]. The strategy of thermotolerance by plants is based on activating an initial moderate-temperature increase response to contrast subsequent extreme temperatures. This is done by preventing or repairing the potential damage to heat-labile proteins and membranes [183]. Thermotolerance in plants triggers the induction of sets of heat shock proteins (HSPs), which include the action of chaperones which assists conformational protein folding/unfolding and maintains cellular homeostasis under heat stress [184]. The modification of mechanical properties of the CW represents a key response element to environmental stresses. 

#### 2.6.1. Cell Wall Composition Is Altered in Response to High Temperature

Treatments of plants exposed to 37 °C was reported to generate changes in CW compositions in coffee leaves, which resulted in ∼50% decrease in pectin and 40% increase in hemicellulose components [185]. As an illustrative image, in Chinese cabbage (*Brassica rapa*), it was found that genes related to CW modifications such as XTH, β-glucosidases, expansines, extensins, CesA, glycosyl transferases, pectin esterases, and xylosidases, were upregulated in response to non-lethal temperature treatment (37 °C). The authors suggested that this molecular reprogramming could enable plants to survive to subsequent treatments to extremely high temperatures [186]. It seems that the modulation of plant CWs, a dynamic and heterogeneous polysacharidic matrix, is a key element to induce plant tolerance to environmental stress.

Recently, it was shown that cellulose synthesis-related genes contribute directly to alterations in CW composition in order to maintain CW integrity under environmental stress [164]. In tomato leaves, β-glucosidase, a cellulose hydrolytic enzyme, was shown to be involved in response to heat stress [187]. Similar to what it happens in cereals, in wheat, it was shown that another β-glucosidase could be involved in developing drought-tolerant wheat seedlings by modifying the CW polysaccharide composition and favoring drought tolerance [188]. Under stress conditions, transcriptional reprogramming induced the production of certain CW proteins, affecting the synthesis, deposition, and reorganization of CW-containing polysaccharides affecting CW architecture [189]. Among them, pectins are crucial elements for plant tolerance to high temperatures, as reported in many species such as bromeliad (*Nidularium minutum*), Arabidopsis, oilseed rape (*Brassica napus* var. *oleifera*), and rice [112,190,191,192]. It has been well reported that the DM of pectins is regulated by the action of PMEs in the cytosol. PMEs remove methyl ester groups to modulate intracellular adhesion by inducing connections between demethylesterified pectin domains by crosslinking each other through de novo formation of calcium (Ca^2+^) bonds [30,119,193]. During plant differentiation processes and in response to stress responses the pectin DM influences the wall rigidity and solubility of the CW matrix. Thus, PMEs positively influence tolerance to elevated temperatures by maintaining proper apoplastic Ca^2+^ levels [193]. High temperature activation of PME activity induces the removal of Ca^2+^ bridges from homogalacturonan backbound upon heat stress tolerance by activating the expression of certain HSPs to induce CW remodeling in order to maintain plasma membrane integrity [112]. For example, the Arabidopsis PME34 mutant plants, presented thermotolerance impairment. It has been suggested that PME34 functions in controlling CW mechanical properties in order to modulate stomatal movements and the flexibility of the guard CW necessary to develop a heat response [192]. 

The expansin (EXP) family could also play a crucial role in CW integrity in response to heat stress. In the heat-tolerant plant species *Agrostis scabra, As*EXP1 was strongly induced in response to high temperature. This was associated with thermotolerant grass germplasm [194,195]. In tobacco, the overexpression of a Kentucky bluegrass (*Poa pratensis*) *Pp*EXP1 reduced cell structural damage and enhanced heat stress tolerance [165]. As described before, XTH and XET play a crucial role in the modification of the load-bearing CW framework. It was reported, in Arabidopsis, that many different *XTH* genes display altered transcriptional response to developmental and environmental stimuli such as cold and heat shock, drought, and salt stresses [196,197]. It was shown that dehydration and heat stress controls both XTH expression and XET enzymatic activity in wheat seedlings, and this is directly linked to the degree of tolerance to heat stress displayed by wheat cultivars [198]. 

#### 2.6.2. AGPs Respond to High Temperature Stress

Under high-temperature conditions, several reports have indicated that AGPs expression levels were altered. AGPs are involved in the stiffening of CW by oxidative crosslinking which enables cells to reduce the loss of water caused by high temperatures [199]. In tomato, high temperatures transiently downregulated AGPs in stems [200], whereas an increase in arabinose and galactose polymers content, the main component of AGPs, was reported in the leaves of coffee under heat stress conditions [185]. The authors suggested that the high content of AGPs in leaf CW could contribute by increasing the ability of these plants to survive upon repeated cycles of desiccation and rehydration. In reproductive organs, the expression of AGPs was downregulated mainly in the stigmas and ovules in tomato plants (*S. lycopersicum* cv. Micro-Tom) [124,125]. In stems and reproductive tissues that are not directly exposed to atmospheric conditions, it has been suggested that AGP expression is downregulated to save more energy. However, in leaf tissues, which are more susceptible to water loss, AGP contents increase accompanying an increase in the epidermal CW thickness which would limit further water loss caused by heat stress [124,125]. Overall, currently, the main CW factors (from transcriptional and post-transcriptional regulating factors, to the single enzymes involved) that contribute to the activation of plant thermotolerance remain largely unknown in most plant species, and therefore further investigation is required. 

## 3. Conclusions

In order to predict how plant physiology responds to changing climate, both structural and functional components, such as CW properties, plant metabolism, leaf size, and photosynthesis have to be coordinated for proper C assimilation and incorporation into a functional response model (Figure 1). CW is the most important sink of carbonic resources in plants, therefore, the allocation of C to CW synthesis and the balance of this with starch metabolism must be carefully regulated for optimal growth. There are evidences showing that C status (such as the availability of sugars) is a critical factor controlling the rate of CW synthesis (discussed in Figure 2). The molecular mechanisms underlying this control have yet to be fully explored, although significant progress has been made exploring the translocation of C from source tissues to sink tissues [201,202,203,204]. Global warming affects several environmental conditions affecting plants such as an increase in CO_2_ levels, the appearance of heat, drought, and salinity stresses, and the enhancement of photosynthetic rate in response to these multifactorial effects. The mechanical and compositional conformation of CW is severely affected by abiotic stress, but the way the CW is modulated differs among all types of stresses. In this review, we have focused on the impact of several climate change-induced abiotic stresses on plant CW and summarized most of them.

Changes in CW in response to drought, heat, or salinity have been documented during the last decades, however, there is less data concerning some other stresses such as an increase of CO_2_ and the impact on alterations in C fixation and photosynthetic efficiency. For this paper, we reviewed data available in the literature which were mostly related to transcriptome, biochemical analysis, and functional studies based on disruption of the chemical composition of the CW in response to abiotic stresses. Because responses to climate change are species dependent, there is a genetic contribution to climatic responses depending on plant adaptation (acquired upon evolution) or how they were acquired during plant domestication. We describe many of them in different crops of interest (described briefly in Table 1). 

## 4. Future Directions

As discussed in this review, a close link exists between photosynthesis and CW synthesis (Figure 3). Photosynthesis appeared about three billion years ago and, starting from that period, the atmospheric CO_2_ concentration gradually decreased causing a cooling of the Earth until the industrial revolution when the phenomenon was reversed following the use of huge quantities of fossil fuels. Photosynthesis has continuously removed CO_2_ from the atmosphere, delivering it into the CW. Thus, the role photosynthesis plays in the natural environment, by absorbing atmospheric CO_2_, is critical to mitigating the climate change effects of atmospheric greenhouse gases. More CO_2_ has to be removed from the atmosphere to limit global warming, therefore, biotechnological tools aimed at increasing the photosynthetic rate or the engineering of plants for enhanced CO_2_ fixation are of great interest. Decreased plant biomass under environmental stress is indicative of a reduction in plant fitness and an adaptive response to environmental stress. A significant challenge for the future is to decipher how the biosynthetic pathways that are linked to specific CW components can be manipulated, and how CW components can be coordinated with each other in specific developmental processes to ensure appropriate allocation of C resources to CW biosynthesis. We have described biotechnological advancements that have led to the discovery of key genes that control CW homeostasis and facilitate tolerance to climate change-related stresses in many crops of interest (cereals such as rice, barley, wheat, and maize) and other key dicots (such as brassicas, cotton, tomato soybean, and potato) among others. This information is of great interest for the rational design of genetic engineering traits aimed at increasing stress tolerance in key crops via CW manipulation. Summarizing, a significant lack of understanding makes it difficult to predict the effect of global warming on plant developmental processes. An understanding of how plant differentiation processes and functions (e.g., photosynthesis, CO_2_ assimilation, and CW homeostasis) will respond to climate change is urgently needed in order to meet the dual challenges of 21^st^ century agriculture. First, to sustain human population growth needs and, secondly, to minimize some of the harmful environmental impacts and cope with future global warming scenarios. We propose a dual effort that aims to (i) optimize energetic efficiency to enhance C fixation and (ii) ensure plant tolerance.

## Figures and Tables

**Table 1:** Cell wall modifications discussed in this work that were reported in response to salinity, drought, heat, and elevated CO_2_ stresses.

Stress	Polymers	Species	Gene	Reference
Salinity stress	Cellulose	*Arabidopsis thaliana*	*CesA*	Chen et al., 2005; Zhu et al., 2010 [108,109]
	Pectins	*Arabidopsis thaliana*	*PME*	Yan et al., 2018; Jithesh et al., 2012 [110,111]
		*Arabidopsis thaliana*	*PAE*	Philippe et al., 2017 [114]
		*Glycine max*	*PAE*	Tenhaken, 2015; An et al., 2014 [115,116]
	Hemicellulose	*Arabidopsis thaliana*	*XTH*	Le Gall et al., 2015; Tenhaken, 2015; Cho et al., 2006; Choi et al., 2011; Han et al., 2017 [115,119,120,121,122]
		*Solanum lycopersicum*	*XTH*	Han et al., 2017 [120]
		*Capsicum annuum*	*XTH*	Choi et al., 2011 [121]
		*Nicotiana tabacum*	*XTH*	Han et al., 2014 [123]
	Cell wall proteins	*Arabidopsis thaliana*	*EXP*	Shen et al., 2014; Abuqamar et al., 2013 [117,118]
		*Arabidopsis thaliana*	*EXLA*	Tenhaken, 2015; Abuqamar et al., 2013 [115,118]
		*Arabidopsis thaliana*	*GPRs*	Shen et al., 2014 [117]
		*Arabidopsis thaliana*	*AGPs*	Shen et al., 2014 [117]
		*Nicotiana tabacum*	*AGPs*	Olmos et al., 2017; Zhu et al., 1993; Zhu et al., 2002; Lamport et al., 2006 [68,126,129,130]
		*Oryza sativa*	*AGPs*	Ma & Zhao, 2010 [127]
		*Brassica oleracea*	*AGPs*	Fernandez-Garcia et al., 2011 [128]
		*Arabidopsis thaliana*	*FLA*	Johnson et al., 2011 [131]
		*Populus trichocarpa*	*FLA*	Zang et al., 2015 [132]
Elevated atmospheric CO2	Cellulose	*Hymenaea courbaril*	*CesA*	Aidar et al., 2002 [144]
		*Arabidopsis thaliana*	*CesA*	Teng et al., 2006 [142]
		*Quercus petraea*	*CesA*	Koike et al., 2018 [140]
		*Hymenaea courbaril*	*CesA*	Aidar et al., 2002 [144]
		*Arabidopsis thaliana*	*CesA*	Teng et al., 2006 [142]
	Pectins	*Populus tremuloides*	*PME*	Le Gall et al., 2015 [119]
	Hemicellulose	*Plantago media*	*XET*	Sharma., 2014 [145]
		*Liquidambar styraciflua*	*XTH*	Kim et al., 2015 [146]
		*Populus tremuloides*	*XTH*	Gupta et al., 2010 [151]
		*Betula pendula*	*XTH*	Oksanen et al., 2005 [143]
		*Hordeum vulgare*	*XTH*	Le Gall et al., 2015; Ookawara et al., 2005 [119,149]
	Cell wall proteins	*Hordeum vulgare*	*EXP*	Le Gall et al., 2015; Ookawara et al., 2005 [119,149]
		*Liquidambar styraciflua*	*EXP*	Kim et al., 2015 [146]
		*Populus tremuloides*	*EXP*	Gupta et al., 2010 [151]
Drought stress	Cellulose	*Arabidopsis thaliana*	*CesA*	Chen et al., 2005, Xie et al., 2011 [108,158]
	Pectins	*Arabidopsis thaliana*	*PME*	Wormit & Usadel, 2018; Yang et al., 2019 [113,170]
	Hemicellulose	*Arabidopsis thaliana*	*XTH*	Cho et al., 2006 [122]
		*Solanum lycopersicum*	*XTH*	Choi et al., 2011 [121]
		*Arabidopsis thaliana*	*EXP*	Liu et al., 2019; Dai et al., 2012; Han et al., 2017 [120,163,167]
		*Nicotiana tabacum*	*EXP*	Xu et al., 2014 [165]
		*Glycine max*	*EXP*	Guo et al., 2011 [166]
	Cell wall proteins	*Oryza sativa*	*AGP*	Tseng et al., 2013 [179]
		*Arabidopsis thaliana*	*AGP*	Seki et al., 2002 [178]
Heat stress	Cellulose	*Brassica rapa*	*CesA*	Yang et al., 2006 [186]
	Pectins	*Brassica napus*	*PME*	Yu et al., 2014 [190]
		*Coffea arabica*	*PME*	Lima et al., 2013 [185]
		*Nidularium minutum*	*PME*	Carvalho et al., 2013 [191]
		*Glycine max*	*PME*	Wu et al., 2010 [193]
		*Arabidopsis thaliana*	*PME*	Huang et al., 2017 [192]
	Hemicellulose	*Brassica rapa*	*XTH*	Yang et al., 2006 [186]
		*Triticum aestivum*	*XET*	Iurlaro et al., 2016 [198]
		*Coffea arabica*	*XTH*	Lima et al., 2013 [185]
		*Triticum aestivum*	*XTH*	Iurlaro et al., 2016 [198]
		*Arabidopsis thaliana*	*XTH*	Xu et al., 1996 [197]
	Cell wall proteins	*Brassica rapa*	*EXP*	Yang et al., 2006 [186]
		*Brassica rapa*	*EXT*	Yang et al., 2006 [186]
		*Agrostis scabra*	*EXP*	Xu et al., 2007 [195]
		*Nicotiana tabacum*	*EXP*	Xu et al., 2014 [165]
		*Solanum lycopersicum*	*AGP*	Li and Showalter 1996; Mareri et al., 2016 [54,124]
		*Coffea arabica*	*AGP*	Lima et al., 2013 [185]

## Abbreviations

CW	cell wall
Suc	sucrose
Glu	glucose
Fru	fructose
Fru-6-P	fructose-6-phosphate
CesA	cellulose synthase
CW INV	cell-wall invertase
INV	invertase
SuSy	sucrose synthase
WT	wildtype
PME	pectin methylesterase
PMEI	pectin methylestersase inhibitor
CSL	CesA-like protein
PAE	pectin acetylesterase
GPRs	glycine-rich proteins
AGPs	Arabinogalactan proteins
EXL	expansin-like
XTH	xyloglucan endotransglucosylase
FLA	fasciclin-like arabinogalactan-proteins
FER	feronia
HG	homogalacturonan
RLK	receptors-like kinase
XET	xyloglucan endotransglycosylase
EXP	expansin
GT	galacturonosyltransferase-like
BIN	brassinosteroid insensitive
BR	Brassinosteroid
GH	glycoside hydrolases
UGPase	UDP-glucose pyrophosphorylase
CO_2_	carbon dioxide
C	carbon
UDP	uridine diphosphate glucose
HGRPs	hydroxyproline-rich glycoproteins
GPI	glycosylphosphatidylinositol
HSPs	heat shock proteins
RGI	rhamnogalacturonan I
RGII	rhamnogalacturonan II
XET	xyloglucan endotransglucosylase
MUR4	UDP-D-xylose 4-epimerase
SOS	salt overly sensitive
